# Fungi shape genome evolution of bacteria even in the absence of major growth phenotypes

**DOI:** 10.1093/ismejo/wraf081

**Published:** 2025-05-03

**Authors:** Emily E Putnam, Robert May, Nina Freeman, Dillon Arrigan, Andrew Boylan, Laura H Childs, Benjamin E Wolfe

**Affiliations:** Department of Biology, Tufts University, Medford, MA 20155, United States; Department of Biology, Middlebury College, Middlebury, VT 05753, United States; Department of Biology, Tufts University, Medford, MA 20155, United States; Department of Biology, Tufts University, Medford, MA 20155, United States; Department of Biology, Tufts University, Medford, MA 20155, United States; Department of Biology, Tufts University, Medford, MA 20155, United States; Department of Biology, Tufts University, Medford, MA 20155, United States; Department of Biology, Tufts University, Medford, MA 20155, United States

**Keywords:** bacterial-fungal interactions, cheese rind, experimental evolution, genome evolution, *Geotrichum*, *ntrB*, *Penicillium*, *Pseudomonas*, *RNA-seq*

## Abstract

Studies of microbial interactions often emphasize large, easily measurable growth differences and short-term ecological outcomes spanning just a few generations. However, more subtle interactions, such as those without obvious phenotypes, may play a significant role in shaping both the short-term ecological dynamics and the long-term evolutionary trajectories of microbial species. We used the cheese rind model microbiome to examine how two fungal species, *Penicillium camemberti* and *Geotrichum candidum*, impact global gene expression and genome evolution of the bacterium *Pseudomonas carnis*. Even though fungi had limited impacts on the growth of *P. carnis*, ~4–40% of its genome was differentially expressed, depending on the specific fungal partner. When we evolved this *Pseudomonas* strain alone or in co-culture with each of the fungi, we observed frequent mutations in global regulators of nitrogen regulation, secondary metabolite production, and motility, depending on the fungus. Strikingly, many strains with mutations in the nitrogen regulatory gene *ntrB* emerged when evolved alone or with *G. candidum*, but not with *P. camemberti*. Metabolomic and fitness experiments demonstrate that release of free amino acids by *P. camemberti* removes the fitness advantages conferred by *ntrB* mutations. Collectively, these results demonstrate that even in the absence of major short-term growth effects, fungi can have substantial impacts on the transcriptome and genomic evolution of bacterial species.

## Introduction

Through a combination of microbiome sequencing surveys, experimental approaches, and models, a growing number of studies have characterized the diversity and impacts of microbial interactions within microbiomes [1–6]. From highly synthetic communities in the lab to more complicated natural communities in the wild, these studies have demonstrated how interactions among microbial species can have substantial fitness consequences for the interacting species [7–10] and how microbial interactions can affect the larger structure and function of microbiomes [11–15]. Genetic and metabolomic approaches have begun to reveal the chemical and physical mediators of microbial interactions [16–18].

Many studies of microbial interactions tend to focus on interactions with large and easy-to-measure differences in growth rate or total growth for at least one interacting microbe [4, 7]. This bias is partly due to the ease of measuring microbial growth or other straightforward phenotypes in interaction experiments [3]. However, microbes may experience strong biotic impacts from neighboring microbes even in the absence of clear changes in growth rate or abundance. For example, Species A might deplete a commonly shared resource more quickly, prompting Species B to shift its metabolism toward using an alternative source. Although this shift may not lead to noticeable changes in the total growth of Species B, it could have profound implications for its biology. Many cryptic interactions that are difficult to measure in the lab may still have important roles in the ecology and evolution of the microbes.

In addition to focusing on microbial interactions with large growth effects, many microbial interaction studies also focus on short-term ecological outcomes over just a few generations of growth [19]. However, microbes are often subject to biotic selective pressures from co-occurring species over hundreds of generations [20–22]. These longer-term interactions are especially likely to occur within systems where microbial communities are regularly supplied with new resources and can grow at relatively constant rates (e.g. chemostats or the human gut) or in systems where new substrates frequently become available for colonization, leading to ongoing community reassembly on those substrates (e.g. food production facilities, the built environment, dung). How microbial interactions shape the longer-term evolution of microbial genomes and phenotypes in these dynamic settings is still largely unexplored.

Using experimental passaging of microbial populations alone or in co-culture, some studies have begun to explore how biotic interactions could shape the evolution of microbes [20, 23–33]. Many of these studies have focused on the evolution of *Pseudomonas fluorescens* in a variety of different biotic environments, demonstrating that biotic interactions can alter the rates and modes of microbial evolution due to reductions in population size, competition for nutrients, and through other mechanisms [27–29, 33]. Even so, these studies generally only focus on a single strain of one bacterial species and do not identify specific mechanisms of the microbial interactions; moreover, the experimental environment does not generally reflect more realistic biotic environments (however, see [28, 34]). Other recent studies have expanded beyond *P. fluorescens* to begin to understand how interspecific interactions affect the evolution of other microbial species [25, 35, 36], but the limited phylogenetic diversity and biotic conditions of these studies constrain our understanding of how microbial interactions affect phenotypic and genomic evolution in naturally forming microbiomes.

**Figure 1:**
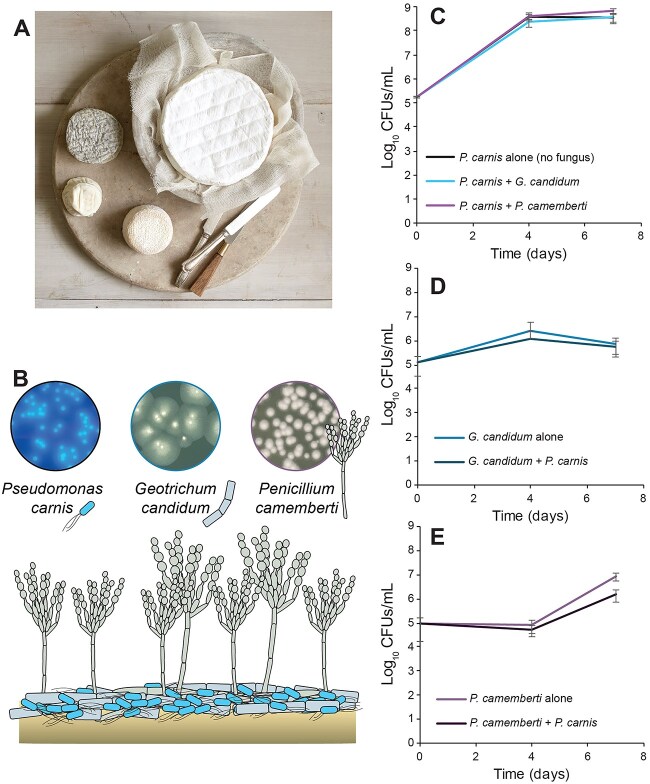
Fungi have a limited impact on the growth of *P. carnis* on CCA. (A) The microbial community we model in this work can be found on bloomy rind cheeses, such as those pictured here. Photograph by Adam DeTour and used with permission. (B) The three microbial species and cheese rind model used throughout this manuscript. Circular photographs illustrate morphological appearances of microbial colonies on LB agar (with blacklight) for *P. carnis* and on PCAMS for *P. camemberti* and *G. candidum*. (C-E) Microbes were grown on pH-adjusted CCA, either alone or in pairs inoculated at a 1:1 ratio, for 4 or 7 days. Absolute abundance was assessed by CFU plating on media supplemented with natamycin or tetracycline. Data represent the average of three independent experiments, each with five biological replicates. Error bars show +/− 1 standard deviation. (C) Abundance of *P. carnis* grown alone, with *G. candidum*, or with *P. camemberti*. *P. carnis* has a small but significant increase in growth in the *P. camemberti* treatment compared to the alone and *G. candidum* treatments after 7 days of growth (ANOVA *F*_(2,44)_ = 17.4, *P* < 0.001). (D) Abundance of *G. candidum* grown alone or with *P. carnis*. *P. carnis* did not affect the growth of *G. candidum* (ANOVA *F*_(1,29)_ = 1.5, *P* = 0.24). (E) Abundance of *P. camemberti* grown alone or with *P. carnis*. *P. carnis* had a significant, but small negative impact on the growth of *P. camemberti* (ANOVA *F*_(1,29)_ = 73.7, *P* < 0.001).

Cheese rind microbiomes are useful systems to explore mechanisms of microbial interactions and bacterial adaptation in different biotic environments. Cheese rinds form on the surfaces of naturally aged cheeses such as Camembert (Fig. 1A) and contain a relatively low diversity microbial community with bacteria, yeasts, and molds [37–39]. Several previous studies have demonstrated that fungi can strongly impact the biology of co-occurring bacterial species [11, 12, 17, 40–43], but the mechanisms underlying these interactions are not well-characterized. Moreover, most of the previous work on the ecology of cheese rind microbial interactions has focused on short-term outcomes and has not considered how interactions with cheese rind fungi could shape the evolution of cheese rind bacteria [23, 25].

*Pseudomonas* species in the *P. fluorescens* group are common contaminants of cheeses, where they can cause aesthetic and flavor defects [44–47]. They are often found in cheeses with a bloomy rind, such as Brie or Camembert, where they grow with the fungi *Penicillium camemberti* and *Geotrichum candidum* (Fig. 1B) [37]. *Pseudomonas* has been shown to benefit from growth with fungi in cheese rinds [37, 40], but little is known about how these short-term fungal-*Pseudomonas* interactions impact the biology of *Pseudomonas* in cheese rinds.

Given the long history of fluorescent pseudomonads as models for studying microbial evolution [48–50] and the limited understanding of the ecology of *Pseudomonas* in cheese rinds, we used a fluorescent *Pseudomonas* isolate (*Pseudomonas carnis* strain LP) to measure how fungi impact the growth, transcriptome, and genome evolution of bacterial species. *P. carnis* is one of many species in the *P. fluorescens* group that contaminate cheeses and other foods [51–53], but its ecology and evolution is almost completely unknown. Because *P. camemberti* and *G. candidum* can alter the abiotic environment of cheese through decomposition of milk proteins [54], we predicted that *P. carnis* would have strong growth and transcriptomic responses to these common cheese fungi. We also predicted that experimental evolution of *P. carnis* alone and with these fungi would select for genomic and phenotypic diversity, depending on the biotic environment.

## Material and methods

### Strains and culture methods

A fluorescent *Pseudomonas* strain (*Pseudomonas carnis* strain LP) was isolated from the rind of a raw milk cheese produced in the United States. To provide a taxonomic assignment of this isolate, we compared the ANI values of our isolate to reference genomes of closely related species that were noted upon depositing the genome in NCBI. These included *Pseudomonas lactis* (strain DSM 29167; NCBI WGS JYLO00000000), *P. paralactis* (strain DSM 29164; NCBI WGS JYLN00000000), and *P. carnis* (strain B4–1; NCBI WGS CABIVL000000000) [53]. ANI values (calculated using the ANI calculator http://enve-omics.ce.gatech.edu/ani/index) between *P. carnis* strain LP and other species are 90.3% for *P. paralactis,* 94.7% for *P. lactis*, and 99.0% for *P. carnis*. Because an ANI of 95% or higher is often used for assigning a species name to bacterial isolates, we call this isolate *P. carnis* LP (hereafter referred to as *P. carnis*).

The fungal strains *Geotrichum candidum* 242A and *Penicillium camemberti* SAM (hereafter *G. candidum* and *P. camemberti*) were previously isolated from cheese [37]. Strains were grown on cheese curd agar (CCA) containing 10% lyophilized cheese curd, 0.5% xanthan gum, 3% NaCl, and 1.7% agar [55]. Unless otherwise noted, CCA was pH-adjusted with a final concentration of 0.0165 M NaOH to a pH of 5.13, as non-pH-adjusted CCA is too acidic to allow consistent growth of *Pseudomonas* strains. Strains were cultured from frozen glycerol stocks (15% v/v glycerol) that had been previously plated out to determine the concentration of viable cells.

CFUs were plated and quantified using plate count agar with milk and salt (PCAMS; 5 g/L tryptone, 2.5 g/L yeast extract, 1 g/L dextrose, 1 g/L whole milk powder, 10 g/L NaCl, and 15 g/L agar) [55] or LB agar as indicated. Tetracycline was added at a final concentration of 15 μg/ml, as needed to inhibit bacteria growth and allow quantification of fungal CFUs. Natamycin was added at a final concentration of 21.6 mg/L, as needed to inhibit fungal growth and allow quantification of bacterial CFUs.

All CFU counting plates and experimental units were incubated at 24°C for the indicated time period.

### Bacterial whole-genome sequencing

*P. carnis* genomic DNA was isolated using the QIAgen DNeasy Blood & Tissue Kit (QIAgen 69 556, Germantown, MD, USA) per the manufacturer’s instructions. Samples were shipped to the Microbial Genome Sequencing Center (MiGS, Pittsburgh, PA, USA; now known as SeqCenter) for sequencing on a NextSeq 2000 (Illumina) with paired-end, 151 base-pair reads. Demultiplexing, quality control, and adapter trimming was performed using bcl2fastq (2.20.0.445). Sequencing reads were assembled using SPAdes (v3.15.3), annotated using RASTtk (v1.073), and contigs were concatenated using Geneious Prime (version 2022.1.1).

### Interaction assays

To understand how *P. carnis* interacts with other members of the cheese rind community, we assessed growth of the bacterium either grown alone or combined with *G. candidum* or *P. camemberti* at a 1:1 CFU ratio. The mixture was added to 1.5 ml microcentrifuge tubes containing 150 μl CCA (pH adjusted), for a final cell concentration of 1000 CFUs/strain/microcentrifuge tube (the equivalent of 25 CFUs/strain/mm^2^). Each tube was sealed with an AeraSeal (Millipore Sigma A9224, Massachusetts, MA, USA) and incubated at 24°C for either 4 or 7 days.

Cells were collected by adding 500 μl of 1X phosphate-buffered saline (PBS) to each tube and by pestling the PBS and CCA mixture to make a homogeneous suspension of the solid CCA media and cells. The pestled homogenate was then serially diluted and plated for CFUs. Each time point represents a separate set of samples, as this was destructive sampling.

Interaction assays were conducted with five biological replicates per condition per time point. Results from three independent experiments were averaged. ANOVAs on log transformed total CFUs were used to determine significant differences in microbial abundances across the different interaction treatments.

### RNA-seq of *P. carnis* in different biotic environments

To characterize whether the presence of the two fungi influences aspects of *P. carnis* biology beyond growth, we grew *P. carnis* alone, with *G. candidum*, or with *P. camemberti* on pH-adjusted CCA plates (100 mm standard petri dishes) in an RNA-seq experiment. Each plate was inoculated with ~144 000 CFUs per plate per strain (25 CFUs/strain/mm^2^). This is the same density of cells per area used in the smaller format microcentrifuge tubes for the interaction assays above. We used the larger format CCA setup for RNA-seq experiments to maximize the amount of high-quality RNA that we could obtain for RNA-seq. PBS (1X) was used as a loading control to ensure an equal volume of liquid was spread across each plate. Plates were incubated at 24°C for 4 days. There were four replicate RNA-seq samples generated for each interaction treatment.

After 4 days, a 4 mm plug was removed from the plate to quantify CFUs of *P. carnis*, *G. candidum*, and/or *P. camemberti*. This was done by pestling the plug in 500 μl 1X PBS to homogeneity, serially diluting, and plating on PCAMS with tetracycline to count fungal CFUs or natamycin to count bacterial CFUs.

The cells on the remainder of the pH-adjusted CCA plate were harvested by scraping a sterile razor across the surface of the plate and resuspending cells in RNAprotect (QIAgen). RNA was harvested using a standard phenol chloroform extraction protocol as described previously [55]. Briefly, harvested cells were centrifuged, RNAprotect supernatant was removed, and cells were resuspended in 500 μl 2x Buffer B (200 mM NaCl, 20 mM EDTA), 210 μl 20% SDS, and 500 μl phenol chloroform isoamyl alcohol (125:24:1, pH 4.5) along with sterile beads. Bead-beating was performed using a vortex multi-adaptor for 2 minutes, samples were spun at 8000 rpm for 3 minutes at 4°C, and the aqueous phase was transferred to a fresh tube. 70 μl 3 M sodium acetate and 700 μl ice-cold isopropanol were added and the samples were incubated at −20°C for 1 hour. Samples were then spun at 13 000 rpm for 10 minutes at 4°C to pellet the RNA and washed with 70% ice-cold ethanol. The pellets were air-dried prior to resuspension in nuclease-free water.

RNA was subsequently treated with Turbo DNase (Invitrogen) and cleaned using the Zymo RNA Clean & Concentrator 25 kit per the manufacturer’s instructions (Zymo).

Treated and cleaned RNA samples were shipped to MiGS for additional DNase treatment with Invitrogen DNase, stranded library prep using Illumina’s Stranded Total RNA Prep Ligation, and rRNA depletion using custom probes designed against the rRNA of *P. carnis* strain LP, *G. candidum*, and *P. camemberti*. Paired-end sequencing was performed on a NextSeq 2000 (Illumina) with 2x51 bp reads. Demultiplexing, quality control, and adapter trimming was performed with bcl2fastq (2.20.0.445).

Transcriptomic sequencing data was analyzed using the Geneious Prime (version 2022.1.1). Briefly, sequences were aligned to the *P. carnis* LP reference genome (assembled, annotated, and concatenated as described above) using the Geneious RNA Mapper with medium-low sensitivity settings. Expression levels were compared between samples using DESeq2. We considered differentially expressed genes to be those with greater than two-fold change in expression when comparing growth in the presence of a fungal interacting partner to growth alone. Differentially expressed genes were considered statistically significant with a false discovery rate corrected *P* value of < 0.05.

KOBAS pathway enrichment [56] was used to identify the extent to which specific metabolic pathways were represented among the differentially expressed genes in each condition. Specifically, multi-fasta files of all genes that were significantly up- or down-regulated in each condition were compared to the *P. carnis* strain LP genome, using information from the KEGG database, to determine which pathways were up- or down-regulated in each experimental condition.

### Experimental evolution of *P. carnis* in different biotic environments

To determine how fungal environments can impact genome evolution of *P. carnis*, we grew the bacterium either alone (no fungus) or combined with *G. candidum* or *P. camemberti* at a 1:1 ratio in an evolution experiment. The mixture was added to 1.5 ml microcentrifuge tubes containing 150 μl CCA (pH adjusted), for a final concentration of 1000 CFUs/strain/microcentrifuge tube (25 CFUs/strain/mm^2^). Tubes were sealed as described above and incubated at 24°C for 7 days.

After 1 week of incubation, 500 μl 15% glycerol in 1X PBS (v/v) was added to each tube and pestled to homogenize. 20 μl of homogenized culture and CCA was transferred to a fresh 1.5 ml microcentrifuge tube containing 150 μl pH-adjusted CCA, which were then sealed as described above and incubated at 24°C for 1 week. The remaining homogenized culture was frozen at −80°C as a “fossil record.”

Transfer of cultures to a fresh 1.5 ml microcentrifuge tube containing 150 μl pH-adjusted CCA were conducted weekly for a total of 12 weeks. “Fossil records” were saved weekly. Every 3 weeks, 20 μl of the homogenized culture was also serially diluted and plated for CFUs on PCAMS with tetracycline or natamycin as needed, to quantify growth of *P. carnis* and its fungal interacting partners over time.

At the end of the 12 weeks, cultures were serially diluted and plated for individual colonies. From each biological replicate, 10 individual colonies were chosen using a random grid. Each colony was then used to inoculate an overnight culture in LB broth (24°C, 225 rpm). Overnight cultures were used both to make 20% glycerol stocks of each isolate (frozen at −80°C) and to extract genomic DNA using the QIAgen Blood and Tissue Kit (QIAgen 69 556, Germantown, MD, USA) as per manufacturer’s instructions. Genomic DNA was quantified by NanoDrop and submitted for sequencing at MiGS using a NextSeq 2000 (Illumina) with paired-end, 151 bp reads. Demultiplexing, quality control, and adapter trimming were performed by bclfastq (2.20.0.445).

Sequences were analyzed using the GeneiousPrime software package (version 2022.1.1). Reads were mapped to the *P. carnis* strain LP reference genome using the “Map to Reference” tool and the Geneious mapper with medium/fast sensitivity. Variants were called using the “Find Variations/SNPs” tool when there was at least 20x coverage and when reads detected a variant with at least 90% frequency. We used PERMANOVA (conducted in Past version 4.01) on the full set of non-synonymous mutations (including singletons) to test for significant differences in the types of mutations that were detected across the three biotic conditions. Although singletons were included in the statistical analysis, singletons were excluded from figures for ease of visualization; the complete dataset, including singletons, can be found in Table S1.

### Growth curves of *P. carnis* Ancestor and *ntrB* mutants

To determine how *ntrB* mutations impacted growth of *P. carnis*, we conducted growth experiments with *P. carnis* LP ancestral strain (Ancestor) and evolved *ntrB* point mutant strains. For these fitness assays, we selected two evolved strains, each with one missense mutation in the *ntrB* gene: 9E (isolated from the alone treatment) with an L127P point mutation, and 16C (isolated from the *G. candidum* treatment) with an H73P mutation. These strains were chosen for low levels of other mutations elsewhere in the genome (Table S1, Table S2) to reduce the possibility of off-target effects from other mutations. Although *ntrB* point mutations across all isolates were seen in both the histidine kinase domain (predicted at 462-1086 bp) and the upstream region (which may be the sensor domain) (Table S1), both isolates 9E and 16C had point mutations outside of the predicted histidine kinase domain (see Results below).

Strains were inoculated into 1.5 ml microcentrifuge tubes containing 150 μl pH-adjusted CCA at 1000 CFUs per tube (~25 CFUs/mm^2^). Tubes were sealed as described above and incubated at 24°C for 7 days in a closed tube rack with a paper towel soaked in 10 ml deionized water to maintain humidity. Harvests occurred at 24, 48, 72, and 168 hours after inoculation; a separate set of samples was harvested at each timepoint because sampling was destructive. To harvest, 500 μl 1X PBS was added to each tube and homogenized with the CCA by pestling; the solution was then plated for CFUs.

To determine how amino acid supplementation affected bacterial growth, we used the same procedure as above, except strains were inoculated on pH-adjusted CCA with 1X Gibco MEM free amino acids solution and harvested at 168 hours after inoculation. The 1X amino acid solution contains supplemental amino acids (126.4 mg/L L-arginine hydrochloride, 24.0 mg/L L-cystine, 42.0 mg/L L-histidine hydrochloride-H_2_O, 52.4 mg/L L-isoleucine, 52.4 mg/L L-leucine, 72.5 mg/L L-lysine hydrochloride, 15.1 mg/L L-methionine, 33.0 mg/L L-phenylalanine, 47.6 mg/L L-threonine, 10.2 mg/L L-tryptophan, 36.0 mg/L L-tyrosine, and 46.8 mg/L L-valine) and was added to the CCA to simulate release of amino acids by fungal metabolism. ANOVAs were used to determine differences in total growth of *P. carnis* across the different strains or nutrient conditions.

### *P. carnis ntrB* mutant RNA-seq

To determine how *ntrB* mutations impacted the biology of *P. carnis,* we used the same RNA-seq protocol described above, but with the *P. carnis* ancestral strain and strains 9E and 16C grown alone on CCA. RNA extraction, library preparation and sequencing, and data analysis were conducted as described above. There were four replicate RNA-seq samples generated for each strain.

### *P. carnis ntrB* mutant competition assays

To determine the fitness of *ntrB* mutants in different biotic environments relative to the Ancestor strain, we conducted competition experiments with the Ancestor strain and *ntrB* mutant strain 9E. This strain was selected for competition experiments because it had a smaller colony size after 3 days of growth on LB agar compared to the Ancestor, so mutant and wild type colonies could be easily distinguished (see Fig. S1). We inoculated the experiment with a higher frequency of the Ancestor (~80% Ancestor to 20% 9E) to mimic conditions in the evolution experiment where mutants initially occur at a low frequency.

The mixture of two *P. carnis* strains was then inoculated into 1.5 ml microcentrifuge tubes containing 150 μl pH-adjusted CCA, alone or with either *G. candidum* or *P. camemberti*, for a final concentration of 1000 CFUs/strain/tube (~25 CFUs/strain/mm^2^). Tubes were sealed as described above and incubated at 24°C for 7 days in a closed tube rack with a paper towel soaked in 10 ml deionized water to maintain humidity.

After 1 week of incubation at 24°C, 500 μl 15% glycerol in 1X PBS (v/v) was added to each tube and pestled to homogenize. The homogenate was used for serial dilution and CFU plating on LB with natamycin at 21.6 mg/L to count the Ancestor and mutant CFUs and on PCAMS with tetracycline at 15 μg/ml to confirm continued fungal growth. Ancestor and 9E colonies were distinguished based on size, with 9E colonies having consistently smaller colonies after 3 days of incubation on LB. To confirm that colony size consistently differentiated Ancestor and 9E colonies, we isolated DNA from randomly selected colonies (10 of Ancestor and 8 of 9E) from output plates from one experimental replicate and sequenced the *ntrB* gene. We had 100% accuracy of differentiating the two genotypes by colony size (Fig. S1).

### Free amino acid metabolomics on cheese curd agar

To determine the amount of free amino acids in cheese curd agar, pH-adjusted CCA plates were inoculated with ~144 000 CFUs per plate (25 CFUs/mm^2^), using either *G. candidum* or *P. camemberti,* or 100 μl of sterile 1X PBS as a control. Plates were incubated 24°C for 7 days and shipped to Creative Proteomics for free amino acid analysis. At Creative Proteomics, a methanol:chloroform extraction, derivatization with Water AccQTag, and HPLC with external standards curves were used to detect and quantify amino acids.

## Results

### *G. candidum* and *P. camemberti* have a limited impact on the growth of *P. carnis*

We saw limited effects of both fungi on the growth of *P. carnis* (Fig. 1C), with 0.1-fold higher growth of *P. carnis* in the presence of *G. candidum* and 1-fold higher growth in the presence of *P. camemberti. P. carnis* total CFUs were only significantly higher than the *P. carnis* alone treatment when compared to growth with *P. camemberti* (Fig. 1C). Similarly, the bacterium had little impact on the growth of either fungus; both fungi grew slightly less in the presence of *P. carnis*, with −0.3-fold change for *G. candidum* and −0.8-fold change for *P. camemberti* (Fig. 1D, Fig. 1E). Only growth of *P. camemberti* with *P. carnis* was significantly different from the *P. camemberti* alone treatment (Fig. 1E). These interaction assays demonstrate that *P. carnis* can co-occur with common bloomy rind fungi, and there are minor growth effects resulting from their interactions.

### Fungi differentially impact the transcriptome of *P. carnis*

**Figure 2:**
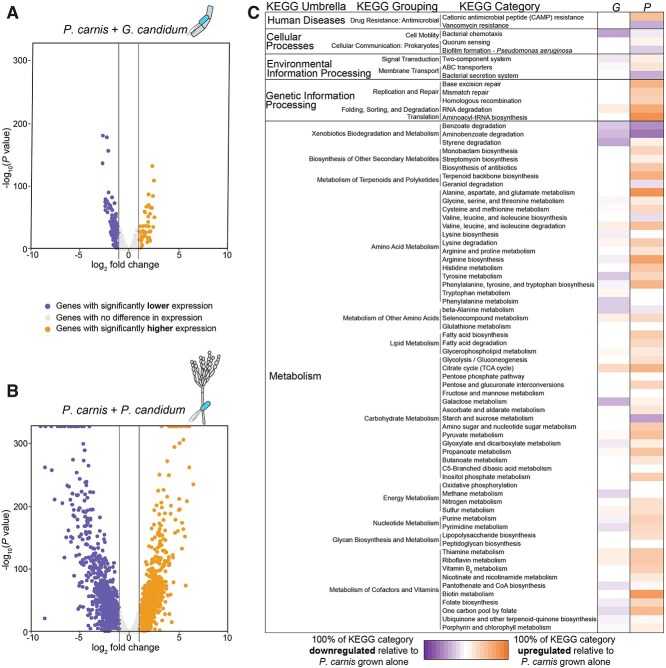
Fungi have divergent impacts on the *P. carnis* transcriptome. *P. carnis* was grown alone, with *G. candidum*, or with *P. camemberti*. (A-B) Volcano plots showing the overall transcriptional response of *P. carnis* to each of the fungi. Orange dots represent genes upregulated by >2-fold with *P* < 0.05. Purple dots represent downregulated genes with the same cutoffs. In B, the dots along the top of the graph indicate genes that had very low *P* values and would have been too high on the graph to be effectively visualized. We plotted them all at the upper bounds for P values on the graph. (C) Percentage of genes in various KEGG categories that were differentially expressed by *P. carnis* with either *G. candidum* (G) or with *P. camemberti* (P). The pathways were determined using KOBAS to assign genes with >1 log_2_-fold change and *P* value <0.05 to KEGG pathways.

RNA sequencing revealed that the two fungi differentially impacted the transcriptional profile of *P. carnis* relative to the transcriptome of *P. carnis* when grown alone. When *P. carnis* was grown with *G. candidum*, 172 genes (2.8% of the predicted 6138 genes in the genome) had significantly decreased expression and 55 genes (0.9% of the genome) had significantly increased expression (Fig. 2A, Table S3). The overall transcriptomic response of *P. carnis* to *P. camemberti* was stronger, with 1297 genes (21% of the genome) having significantly decreased expression and 1243 genes (20% of the genome) having significantly increased expression (Fig. 2B, Table S3).

Pathway enrichment analysis using KOBAS [56] identified several metabolic categories in *P. carnis* that changed in expression in the presence of the fungi (Fig. 2C, Table S4). Two KEGG categories were enriched in genes that increased in expression in the presence of *P. camemberti*: biosynthesis of secondary metabolites and one of its sub-categories, biosynthesis of antibiotics (Fig. 2C, Table S4B). Although no other pathways were significantly enriched, substantial portions of KEGG categories were up- or down-regulated in *P. carnis* in the presence of *G. candidum* or *P. camemberti*, suggesting biological responses by *P. carnis* to its specific neighbor. For example, genetic information processing, including DNA replication and repair, was upregulated in the presence of *P. camemberti* (Fig. 2C, Table S4B)*.* However, this pathway had little transcriptional change in the presence of *G. candidum* (Fig. 2C, Table S4A)*.* Metabolism of cofactors and vitamins was also upregulated only in the presence of *P. camemberti* but not *G. candidum*. Finally, amino acid metabolism was upregulated in the presence of *P. camemberti*, but either experienced no change in transcriptional level or was downregulated in the presence of *G. candidum* (Fig. 2C, Table S4). These broad categories suggest differences in how these two fungal species impact the surrounding environment in which *P. carnis* grows.

Of the pathways that were upregulated in *P. carnis* only in the presence of *P. camemberti*, four KEGG categories had more than 50% of the predicted genes in that pathway upregulated, and all were amino acid metabolic pathways: alanine, aspartate, and glutamate metabolism (84% of the predicted genes in the pathway upregulated); arginine biosynthesis (73%); phenylalanine, tyrosine, and tryptophan biosynthesis (57%); and valine, leucine, and isoleucine degradation (50%) (Fig. 2B, Table S4). Amino acids are a critical nitrogen source for cheese microbes and are largely bound in the polypeptide chains of casein, the main protein in cheese [57, 58]. Proteolysis of casein by fungal extracellular proteases can increase the availability of free amino acids [58–60]. *P. camemberti* has been shown to be able to release more free amino acids from casein compared to *G. candidum* [61], and amino acid metabolic pathways in *P. carnis* were only upregulated in the presence of the more proteolytic *P. camemberti*.

### *P. carnis* evolves unique mutational profiles in different fungal environments

**Figure 3:**
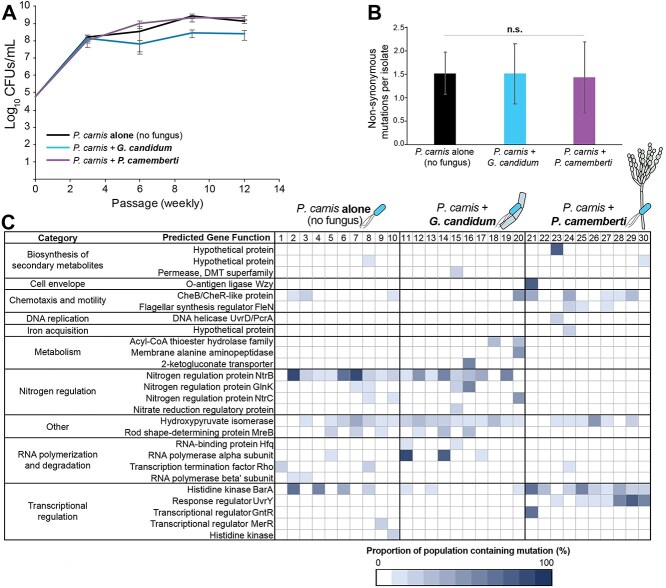
Biotic environments select for different mutations in the *P. carnis* genome*. P. carnis* was grown alone, with *G. candidum*, or with *P. camemberti* on pH-adjusted CCA and passaged onto fresh pH-adjusted CCA weekly for 12 weeks. Each condition was passaged with 10 biological replicates. After the final passage, 10 isolates were selected randomly from each biological replicate for whole genome sequencing, for a total of 300 isolates. (A) *P. carnis* persisted in co-culture with both *P. camemberti* and *G. candidum*. CFUs represent an average across all 10 biological replicates at the indicated time point. Error bars show +/− 1 standard deviation. Final abundance of *P. carnis* at week 12 was slightly significantly higher with *P. camemberti* and significantly lower with *G. candidum* (ANOVA *F*_(2,29)_ = 79.6, *P* < 0.001). (B) Averages are shown for the number of nonsynonymous mutations in coding regions. Error bars indicate +/− 1 standard deviation. (ANOVA *F*_(2,27)_ = 0.1, *P* = 0.94). (C) Heatmap showing nonsynonymous mutations from all 300 replicates, excluding singletons. Numbers across the top correspond to the numbers for each biological replicate. Shade of blue indicates the number of isolates (out of a total of 10) with a mutation in the specified gene for that biological replicate. Only genes with >1 nonsynonymous mutation across the entire experiment are shown. Gene categories were assigned manually. The effect of biotic environment on the frequency of different *P. carnis* mutations was significant (PERMANOVA *F* = 2.2, *P* < 0.001) with the *P. camemberti* treatment being different from alone and *G. candidum* (based on post-hoc Bonferroni-corrected *P*- values), but no difference in mutation frequency between alone and *G. candidum*.

Given that *P. carnis* can grow with either *G. candidum* or *P. camemberti* without major impacts on growth, we were able to serially passage *P. carnis* either with or without a fungus, to determine how different biotic environments affected its genomic evolution. There were an estimated 106, 99, and 109 doublings for the *P. carnis* alone, *P. carnis* + *G. candidum*, and *P. carnis* + *P. camemberti* conditions, respectively, over the course of the 12 weeks. Growth of *P. carnis* remained stable in each condition for the 12-week duration, but there was less total growth and total doublings in the *G. candidum* condition (Fig. 3A). This decrease in *P. carnis* growth was not observed in the short-term experiments completed above (Fig. 1C) and may reflect differences in *P. carnis*-*G. candidum* interactions over longer time scales.

Because the transcriptomic analysis showed upregulation of DNA repair only in the presence of *P. camemberti* (Fig. 2C), we next assessed whether the presence of *P. camemberti*, or any other condition, increased the rates at which mutations accumulated during the experimental evolution. We found an average of 1.5 nonsynonymous mutations per genome, with no significant difference between conditions (Fig. 3B).

Although the overall number of mutations was similar across treatments, the specific type of mutations that we observed were dependent on the biotic environment (Fig. 3C, Table S1). Some mutations, such as those in the gene encoding hydroxypyruvate isomerase, were seen at high rates across all three conditions (nonsynonymous mutations were seen in 12%, 17%, and 11% of *P. carnis* clones from the alone, *G. candidum*, and *P. camemberti* conditions, respectively). Others, such as the mutations seen in the genes encoding the two-component global regulator BarA/UvrY (commonly called GacS/GacA in *Pseudomonas* species), were seen across all three conditions, but the mutations were detected at higher frequencies in the alone and *P. camemberti* conditions (nonsynonymous *barA* mutations were seen in 19%, 3%, and 29% of the *P. carnis* clones from the alone, *G. candidum*, and *P. camemberti* conditions, respectively, and nonsynonymous *uvrY* mutations were seen in 0%, 0%, and 33% of clones from the alone, *G. candidum*, and *P. camemberti* conditions, respectively) (Fig. 3C, Table S1).

One pattern that was apparent across the three biotic environments was the complete lack of mutations in the *P. camemberti* treatment in genes associated with the NtrBC nitrogen regulation system (Fig. 3C, Table S1). When nitrogen levels are low, this two-component signalling system increases the expression of a range of operons across the genome associated with nitrogen assimilation and scavenging [62]. In addition to its key role in regulating nitrogen metabolism, the NtrBC system has also been shown to play roles in motility and virulence [63, 64]. We observed high rates of mutations in the alone and *G. candidum* conditions (34% and 28% of isolates, respectively), but we never observed any *ntrBC* mutations in the *P. camemberti* condition (Fig. 3C, Table S1).

The specific types of mutations often varied from one mutant to the next. For *barA/uvrY*, 32 different kinds of *barA* mutations (including missense, nonsense, 2 bp substitutions, 1 bp insertions, and 1–2 bp deletions) were observed across the 54 isolates with *barA* mutations, and eight different *uvrY* mutations (including missense and 1–2 bp deletions) were observed across the 33 isolates with *uvrY* mutations. For the nitrogen regulatory genes, 17 different missense mutations in *ntrB* were observed across the 89 different isolates with *ntrB* mutations, three different missense mutations in *ntrC* were observed across seven different isolates with *ntrC* mutations, and 4 different missense mutations were observed across the 11 isolates with *glnK* mutations (Fig. 3C, Table S1). Only missense mutations, and no nonsense or frameshift mutations, were observed in any of these three nitrogen regulation genes, despite the prevalence of nonsense and frameshift mutations in other genes such as the *barA*/*uvrY* system.

### *P. carnis ntrB* mutations impact fitness in a context-dependent manner

**Figure 4:**
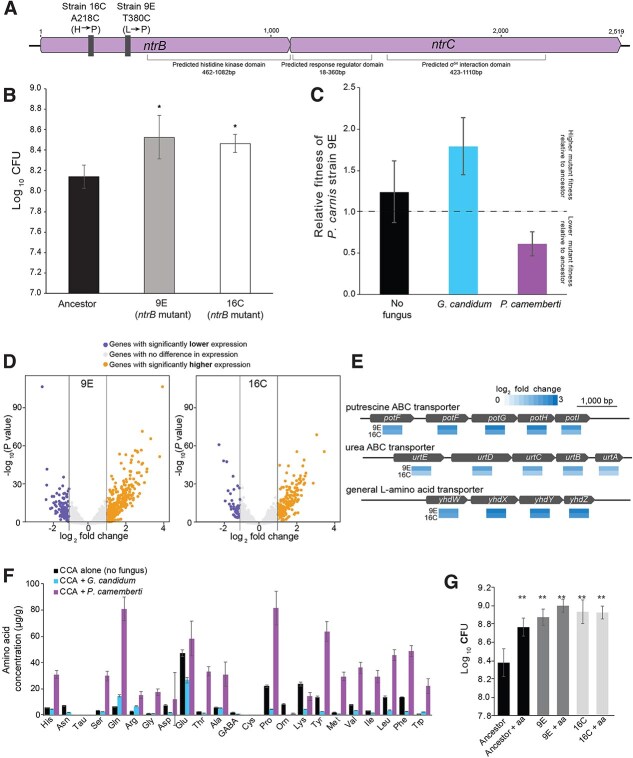
*ntrB* point mutations impact *P. carnis* fitness in a context-dependent manner. (A) Location of SNPs in the *ntrB* gene in the two mutant strains used in experiments (9E and 16C). (B) Growth of the Ancestor *P. carnis* and two *ntrB* mutants (9E and 16C) alone on pH-adjusted CCA demonstrates a higher total growth of mutants (ANOVA F_2,42_ = 29.4; *P* < 0.001). Error bars represent 1 standard error of the mean. N = 3, n = 5. ^*^ indicates significantly different total CFUs compared to the Ancestor based on Tukey’s post-hoc test. (C) Relative fitness of one *P. carnis ntrB* mutant (9E) in three different environments (alone, with *G. candidum,* and with *P. camemberti*). While we plot relative fitness for visual clarity, we used the mean frequency of 9E mutant colonies to determine differences in mutant abundances across each of the three treatments. Mutant frequency was significantly different across the treatments (Kruskal-Wallis test, χ^2^(2) = 50.8, *P* < 0.001; Dunn’s post-hoc test), with a lower mutant frequency (i.e. lower fitness) in the *P. camemberti* treatment compared to the alone and *G. candidum* treatments. Data are a mean of three independent replicates. (D) Volcano plots of RNA-seq data from *ntrB* mutants 9E and 16C show shifts in gene expression. (E) Examples of putative ABC transporter operons that were significantly upregulated in both strains 9E and 16C. Blue shading under each gene indicates log_2_ fold change. (F) Metabolomic data show that fungi, especially *P. camemberti*, increase free amino acid availability. Error bars represent 1 standard error of the mean. n = 3. (G) Supplementing pH-adjusted CCA with amino acids (aa) decreases the growth advantages of the *ntrB* mutants 9E and 16C. N = 3, n = 5. Mean CFUs of the Ancestor strain + aa and all mutant treatments (with and without aa) were significantly higher compared to Ancestor alone (ANOVA *F* = 32.3, *P* < 0.001; ^**^ indicates significant differences among Ancestor and other treatment groups based on Tukey’s post-hoc tests, *P* < 0.001).

Because we observed numerous *ntrB* mutants in the alone and *G. candidum* treatments but never in the presence of *P. camemberti*, we next assessed the fitness of these *P. carnis* mutants (strains 9E and 16C, Fig. 4A) in different biotic contexts. We predicted that *ntrB* mutants would have a higher fitness compared to the Ancestor when cultured alone on pH-adjusted CCA, and that their competitive advantage would be lower in the presence of *P. camemberti* compared to the alone or *G. candidum* treatments.

We first assessed the growth of the Ancestor, 9E, and 16C strains in the absence of any fungi. Both mutant strains grew significantly more on pH-adjusted CCA compared to the Ancestor *P. carnis* (Fig. 4B). 9E showed a 166% increase in growth compared to the Ancestor, and 16C showed a 109% increase in growth compared to the Ancestor. This suggests *ntrB* mutations allow these strains to reach a higher total growth on cheese, possibly due to an ability to utilize resources more efficiently than the Ancestor.

We next assessed the fitness of one *ntrB* mutant strain (9E) in competition with the Ancestor in the three biotic environments from the evolution experiment: alone, with *G. candidum*, or with *P. camemberti*. After 1 week of competition, a significantly lower frequency of 9E colonies were observed in the populations co-cultured with *P. camemberti* compared to the alone and *G. candidum* conditions, suggesting a lower relative fitness of 9E in the *P. camemberti* treatment (Fig. 4C). This aligns with the prediction that the fitness advantage of *ntrB* mutants is lower in the presence of *P. camemberti* and informs the limited abundance of *ntrB* mutants in that condition in the evolution experiment.

### *ntrB* mutations impact the transcriptome of *P. carnis*

To understand the underlying biology of the *ntrB* mutants that might explain their variable fitness in different biotic environments, we used RNA-seq to characterize the transcriptomes of strains 9E and 16C. Compared to the ancestral *P. carnis* strain, the 9E strain showed 313 genes significantly upregulated and 117 genes significantly downregulated (5.1% and 1.9% of the genome, respectively) (Fig. 4D, Table S5). The 16C strain showed 186 genes significantly upregulated and 51 genes significantly downregulated (3.0% and 0.8% of the genome, respectively) (Fig. 4D, Table S5).

Pathway enrichment analysis with KOBAS [56] was used to identify metabolic pathways that were up- or down-regulated (Fig. 4D, Fig. S2, Table S6). Although minor differences were observed between the 9E and 16C strains in the pathways that were up- or down-regulated, the overall trends between the two strains were similar, suggesting that the two different *ntrB* point mutations had similar effects (Fig. 4D, Fig. S2, Table S6). Moreover, the pathway enrichment trends for both 9E and 16C often resembled those observed for the ancestral *P. carnis* grown in the presence of *P. camemberti* (Fig. 2C). Amino acid metabolic pathways were often upregulated, both for the ancestral *P. carnis* in the presence of *P. camemberti*, and also by the 9E and 16C strains relative to the ancestral *P. carnis* grown alone (Fig. 2C, Fig. 4D, Fig. S2, Table S6).

In addition to the broad trends that were revealed by the pathway enrichment analysis, one category in particular was highly significantly upregulated in both mutants: ABC transporters (Fig. 4E, Fig. S2, Table S6). Further investigation of the specific ABC transporters that were upregulated in both mutants included operons for the transport of putrescine (*potFGHI*, [65]), urea (*urtABCDE*, [66]), and general L-amino acids (*aapJQMP,* a.k.a. *yhdWXYZ,* [67, 68]*)* (Fig. 4E, Table S6). All of these transporters are related to the uptake of amino acids and other nitrogen-containing compounds from the extracellular environment and may explain the fitness advantage of the *ntrB* mutants when grown on cheese. Some of these transporters have been found to be regulated by the Ntr system in other bacteria. For example, the proteins encoded by the *aapJQMP* operon can transport a variety of different amino acids in other bacteria [69] and this operon is negatively regulated by the Ntr system in *Rhizobium* [70]. Additional work is required to determine whether these homologs in *P. carnis* operate in the same way as in other bacteria, but this strong signal in our RNA-seq dataset suggests that *P. carnis ntrB* mutants may have increased capabilities to access scarce nitrogen sources in the cheese environment.

### Amino acid availability mediates the fitness advantage of *ntrB* mutants

Given the growth advantages of the *ntrB* mutants and the strong upregulation of amino acid metabolism and nitrogen compound transport observed in the mutants’ transcriptomes, we suspected that the evolution and persistence of the mutations was related to free amino acid availability in the growth media. Specifically, we hypothesized that the *ntrB* mutants have a fitness advantage over the Ancestor strain in cheese environments with lower free amino acid concentrations, such as the alone and *G. candidum* experimental evolution conditions.

To determine the amino acid availability in our RNA-seq and experimental evolution conditions, we used metabolomics to characterize the free amino acid profile of pH-adjusted CCA with no culture, pH-adjusted CCA cultured with *G. candidum*, and pH-adjusted CCA cultured with *P. camemberti*. We observed greater free amino acid concentrations in spent media grown with *P. camemberti* compared to *G. candidum* or the CCA media with no culture (Fig. 4F, Table S7). With *P. camemberti*, the average total concentration of free amino acids was more than 3-fold greater compared to plates with no culture and more than 7-fold greater compared to plates with *G. candidum* growth (Table S7). In particular, we observed increases in free branched-chain amino acids (valine, leucine, and isoleucine), some of the most prevalent amino acids in casein (~20% of the amino acid residues) [71, 72]. With *P. camemberti*, valine levels were more than 4-fold greater compared to plates with no growth and more than 10-fold greater compared to plates with *G. candidum* growth; leucine levels were more than 3-fold greater compared to plates with no growth and more than 12-fold greater compared to plates with *G. candidum* growth; and isoleucine levels were 9-fold greater compared to plates with no growth and more than 18-fold greater compared to plates with *G. candidum* growth (Table S7). These data align with our predictions, as *P. camemberti* can express greater proteolytic activity than *G. candidum* in the cheese environment [73, 74].

To evaluate the effect of elevated free amino acid concentrations similar to those available in the *P. camemberti* condition, we conducted growth experiments of each *P. carnis* mutant strain individually on pH-adjusted CCA and the same media supplemented with free amino acids. Both mutant strains had higher cell concentrations compared to the Ancestor strain (Fig. 4G), matching earlier growth experiments comparing the Ancestor strain to the mutants (Fig. 4B). However, the Ancestor strain grew to a significantly higher concentration on amino acid supplemented pH-adjusted CCA compared to its growth on unsupplemented media; moreover, the Ancestor strain’s growth on amino acid supplemented media was not significantly different from the *ntrB* mutant strains’ growth on amino acid supplemented media (Fig. 4G). In other words, the growth advantage of the mutant strains compared to the Ancestor strain when cultured on standard pH-adjusted CCA was eliminated if the media was supplemented with free amino acids.

**Figure 5:**
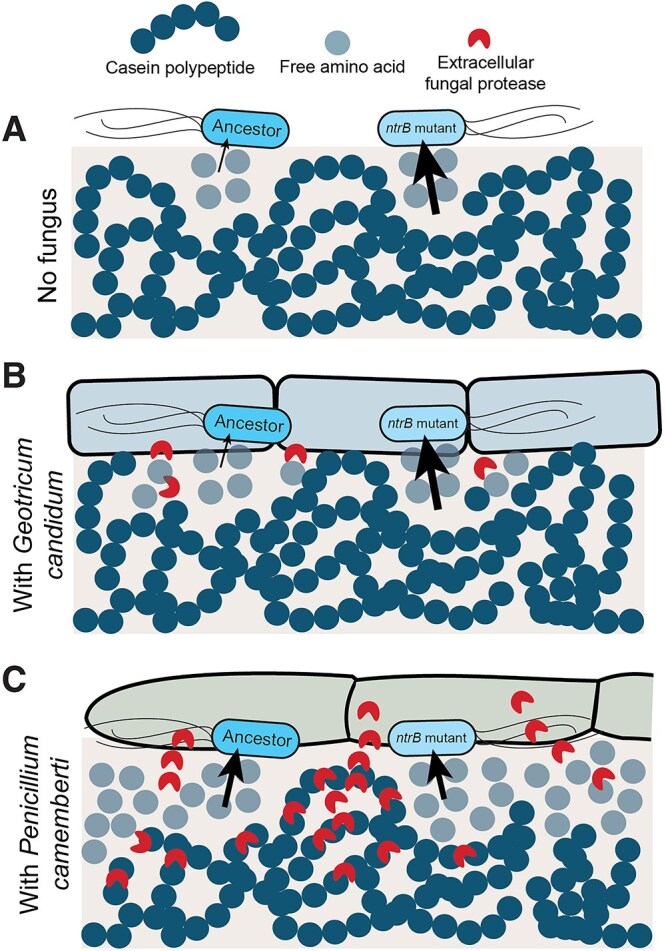
Conceptual model of how fungi alter nutritional environments and associated selective pressures. Without a fungus present in the environment (A) or in the presence of the yeast *G. candidum* (B), the relatively low availability of free amino acids confers a fitness advantage to *P. carnis ntrB* mutants compared to the *P. carnis* Ancestor strain. Mutants have increased expression of amino acid metabolism and nitrogen compound transport pathways that may allow greater utilization of the limited resource. (C) *P. camemberti* produces an abundance of extracellular proteases, increasing the concentration of free amino acids in the environment. Free amino acids are not limited resources in this case, so *P. carnis ntrB* mutants would have no fitness advantage over the *P. carnis* Ancestor strain.

Altogether, our experiments with the Ancestor and *ntrB* mutant strains of *P. carnis* suggest that the mutants’ fitness advantage over the Ancestor is mediated by amino acid availability (Fig. 5). In an environment with limited free amino acid availability (such as monoculture and in co-culture with *G. candidum*), *ntrB* mutants have a significant fitness advantage over the Ancestor strain, which is potentially related to increased expression of amino acid metabolism and nitrogen compound transport pathways. In an environment with comparatively higher levels of free amino acids (such as in co-culture with *P. camemberti* and in media supplemented with free amino acids), *ntrB* mutants have no significant fitness advantage over the Ancestor.

## Discussion

Studies of microbial interactions commonly focus on interactions that have a substantial growth phenotype (e.g. growth rate or absolute growth), where one microbe either enhances or inhibits the growth of another microbe [3, 7, 75]. In this study, *P. camemberti* and *G. candidum* had minimal impacts on the growth of *P. carnis* on cheese curd agar. However, despite the similarities in growth phenotype, *P. camemberti* and *G. candidum* both had large and measurable impacts on the biology of *P. carnis* as observed in both the transcriptomics and experimental evolution assays. Our data are consistent with the idea that non-growth phenotypes, such as changes in transcriptional levels, likely contribute to microbial interactions in ways that are minimal in pairwise interaction assays using solely culture-based methods.

Global regulators are commonly found to explain rapid diversification of bacterial populations in experimental evolution studies [76]. Small genetic changes in a global regulator can alter transcription across many different parts of a bacterial genome and subsequently affect many biological processes [25, 77]. In our evolution assays, we observed parallel evolution of the NtrBC two-component system in *P. carnis*. Past research on this regulatory system has focused on its role in nitrogen metabolism, but NtrBC also plays roles in other biological processes such as biofilm formation and motility [64]. We do not have data at this time that confirms that this nitrogen regulatory system operates in the same way in *P. carnis* as it does in other *Pseudomonas* species, but the strong transcriptional and evolutionary patterns observed here suggest an important role in shaping biological responses to changing nutritional environments. We only characterized two mutants, but we suspect that the range of different types of mutations in *ntrB* created a diverse pool of *ntrB* mutants with different biologies. In addition to *ntrB*, we also observed a range of mutations in genes predicted to encode the BarA/UvrY two-component system in *P. carnis*, also called the GacS/GacA system. In other *Pseudomonas* species, this global regulator controls quorum sensing, metabolite production, and a range of other ecologically significant traits [78, 79]. We did not characterize *barA/uvrY* mutants in this work, but these mutants may have altered fitness on cheese in a similar manner to the *ntrB* mutants. Future work will explore the causes and consequences of *barA/uvrY* mutants in cheese *Pseudomonas* species.

Beyond the evolution of *Pseudomonas* and the Ntr system, our findings from this current study contribute to a common theme in cheese rind microbial ecology research: fungi can shape the ecology of bacterial environments in cheese rinds. Our past short-term ecological studies have noted asymmetrical interactions between bacteria and fungi, where fungi can substantially affect the fitness of bacteria, but bacteria have limited effects on fungi [37]. The mechanisms by which fungi exert these effects is not always determined, but altering resource availability, including iron and free amino acids, is a recurring theme [12, 17]. Even though cheese contains both iron and amino acids, these are typically not easily available to bacteria because they are either chelated (in the case of iron) or locked away in the polypeptide chains of casein (in the case of amino acids) [80–82]. As ecosystem engineers of cheese rinds, fungi can secrete a wealth of metabolites that can alter bacterial growth, gene expression, and evolution. Past studies of different bacteria from the cheese rind system found that fungi could shift iron and amino acid metabolism, often causing downregulation of biosynthesis pathways and upregulation of transport systems [12, 17, 23]. The current work provides another demonstration of how fungi can alter the nutritional environment of cheese and shape the ecology and evolution of rind bacteria. This reaffirms the dominant ecological role of fungi in cheese rinds and points to their use as potential means for cheesemakers to control the ecology and evolution of their microbial foods.

Our model system in the lab attempts to mimic the cheese surface to replicate ecological and evolutionary dynamics in real-world cheeses. However, we acknowledge that the system has some limitations that might make it challenging to directly translate our findings to cheese production systems. First, cheeses rarely contain just two species interacting in simple pairwise scenarios. Many bloomy rind cheeses such as Camembert use both *P. camemberti* and *G. candidum* simultaneously to ripen the cheese and sometimes also include other bacteria to aid with ripening [37, 39, 83, 84]. It is likely that the growth, transcriptomic, and evolutionary changes observed here would change when multiple fungi and other bacteria interact with *P. carnis*. Second, fluorescent pseudomonads are considered undesirable bacteria in cheeses and cheesemakers often work to reduce their abundance in cheese production facilities. Whether a bacterium like *P. carnis* would experience many rounds of growth and population bottlenecks with cheese fungi is not clear. However, *Pseudomonas* species can occur in water lines, surfaces of aging facilities, and raw milk [85, 86], and may therefore constantly invade cheese production systems where they could experience multiple rounds of selection.

The results from our work add to a small but growing body of work that explores when and how microbial interactions can affect the evolution of bacterial species. Work from our own lab and others have shown that interspecific interactions can promote, inhibit, or have limited effects on the rate and mode of bacterial evolution [23–25, 30]. Because there are only a limited number of studies across a small taxonomic diversity of interacting microbes, it is difficult to predict the effects of microbial interactions on microbial evolution. For example, it is reasonable to predict that strong pairwise interactions that affect growth of the target species may have strong impacts on bacterial evolution by altering population sizes, opening new niches, or selecting for traits involved with protection from inhibition by neighboring microbes. However, our data in this study and from past studies across other bacterial-fungal pairings suggest that predicting evolutionary impacts from just growth effects may not be possible. Here we found that very weak ecological interactions over short time scales can have very large evolutionary consequences. In other bacterial-fungal pairings, we found that strong interactions had very weak or limited evolutionary impacts on evolving populations of a *Staphylococcus* species [23]. Additional studies that explore a taxonomically diverse range of interacting pairs and across a range of microbiome environments will hopefully improve our ability to predict the importance of biotic interactions in microbial adaptation.

## Supplementary Material

Combined_Supp_Figures_wraf081

Correct_Combined_Supplemental_Tables_Putnametal_2025_wraf081

## Data Availability

Raw Illumina reads for various datasets in this paper have been deposited in the NCBI SRA with the following BioProject IDs: whole genome re-sequencing reads (PRJNA856811), interaction RNA-seq (PRJNA856809), and *ntrB* mutant RNA-seq (PRJNA915016). The draft genome of *Pseudomonas carnis* strain LP is available in NCBI with the WGS accession # JANCLL000000000.

## Supplementary material

Supplementary material is available at *The ISME Journal* online. Tables S1–S2 (.xlsx) show details for all mutations in the experimental evolution dataset, including singletons. Tables S3–S4 (.xlsx) provide details of the RNA-seq with fungi, including all gene expression values and pathway enrichment results. Tables S5–S6 (.xlsx) show the RNAseq with *ntrB* mutants datasets and pathway enrichment; Table S7 (.xlsx) shows the cheese curd metabolomics data, Fig. S1 (.docx) the validation for different colony sizes between *P. carnis* Ancestor and 9E strains. Fig. S2 (.pdf) shows the heatmap for the dataset associated with Fig. 4D.
