# Supplementary material for: Fungi shape genome evolution of bacteria even in the absence of major growth phenotypes
Source: ISME J. 2025 May 3;19(1):wraf081. doi: 10.1093/ismejo/wraf081 (PMC12411852; doi:10.1093/ismejo/wraf081)
Supplement: Combined_Supp_Figures_wraf081 [file combined_supp_figures_wraf081.pdf]

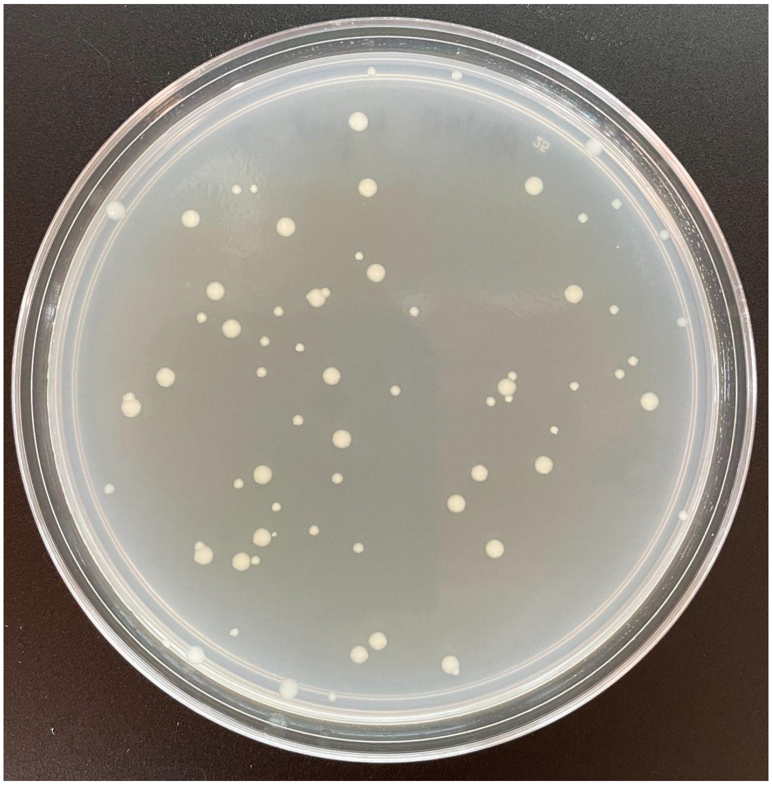

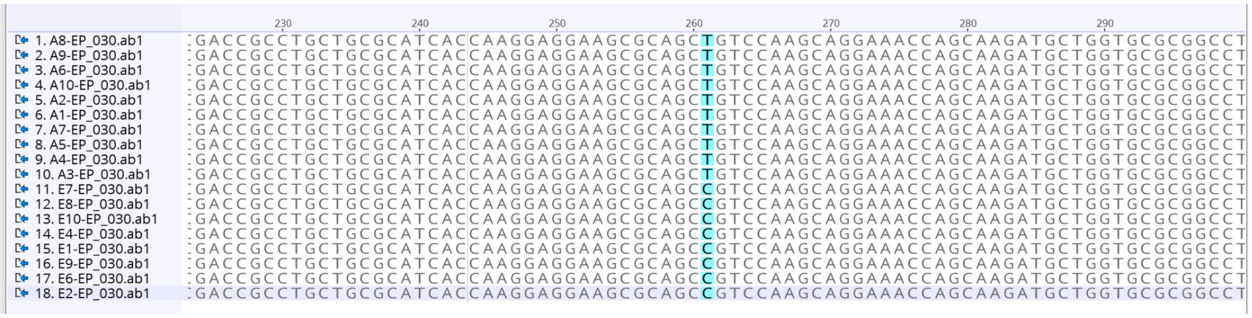

Figure S1: Top image shows differences in colony sizes between *P. carnis* Ancestor (large colonies) and 9E (small colonies) on LB medium after three days of growth at 24 °C. Bottom image shows results from Sanger sequencing colonies from output plates to test whether morphological differences are consistently aligned with sequence differences in the *ntrB* region. We sequenced the *ntrB* region from 10 randomly picked large colonies (Ancestor) and 8 randomly picked small colonies (9E). The zoomed in region shows a single-nucleotide polymorphism that distinguishes the Ancestor *ntrB* sequence (top 10 sequences) from 9E (bottom 8 sequences) and indicates that colony size is a very accurate way to distinguish the two strains in competition experiments.

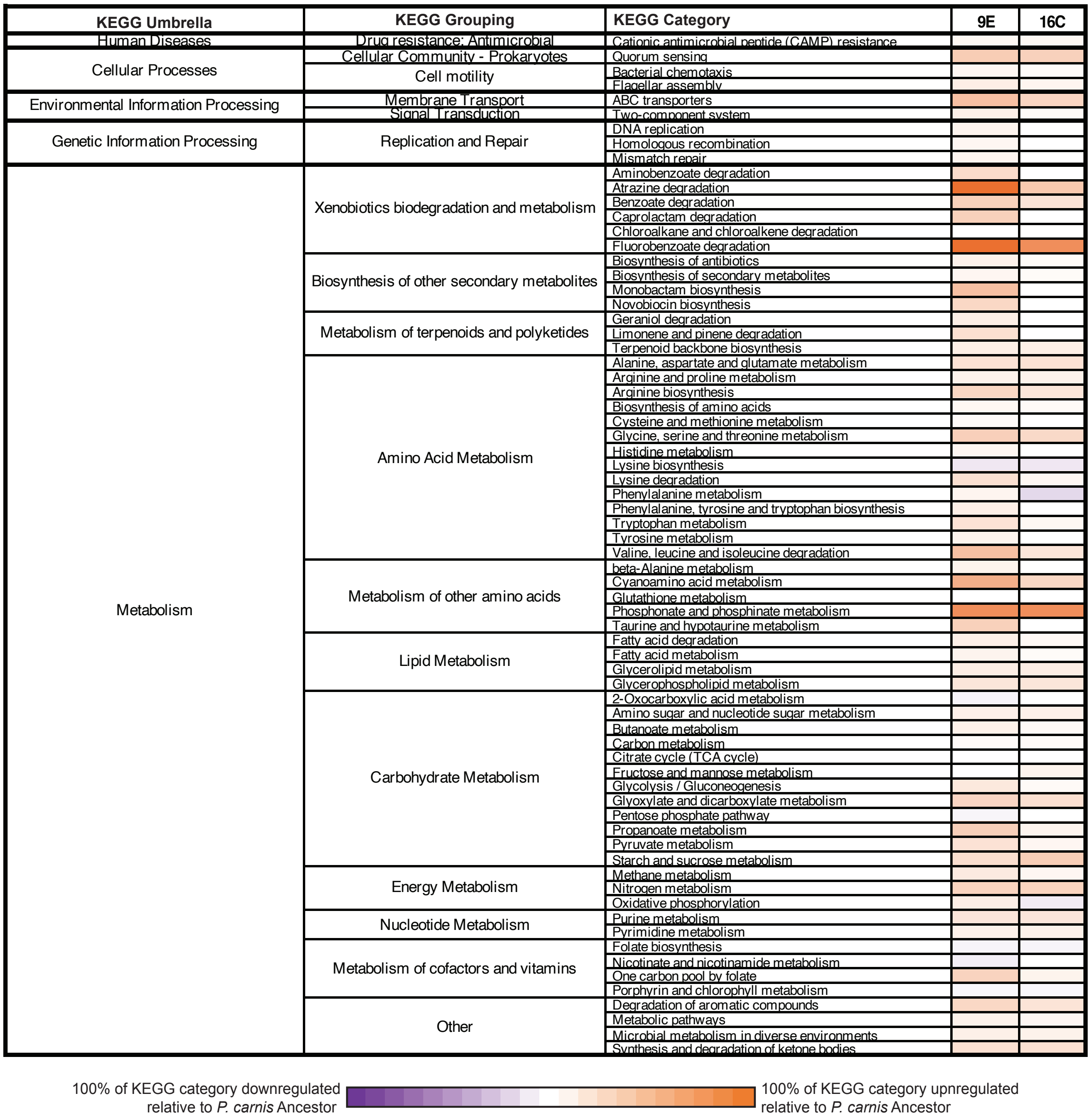

Figure S2: Percentage of genes in various KEGG categories that were differentially expressed by *P. carnis strains 9E and 16C compared to the ancestor strain.* The pathways were determined using KOBAS to assign genes with >1 $\log_2$-fold change and p value < 0.05 to KEGG pathways.
